# Effect of Tai Chi on Quality of Life, Body Mass Index, and Waist-Hip Ratio in Patients With Type 2 Diabetes Mellitus: A Systematic Review and Meta-Analysis

**DOI:** 10.3389/fendo.2020.543627

**Published:** 2021-01-19

**Authors:** Jiawei Qin, Yannan Chen, Shuai Guo, Yue You, Ying Xu, Jingsong Wu, Zhizhen Liu, Jia Huang, Lidian Chen, Jing Tao

**Affiliations:** ^1^ College of Rehabilitation Medicine, Fujian University of Traditional Chinese Medicine, Fuzhou, China; ^2^ Department of Rehabilitation Medicine, Quanzhou First Hospital Affiliated to Fujian Medical University, Quanzhou, China; ^3^ Fujian Key Laboratory of Rehabilitation Technology, Fujian University of Traditional Chinese Medicine, Fuzhou, China; ^4^ Key Laboratory of Orthopedics & Traumatology of Traditional Chinese Medicine and Rehabilitation (Fu Jian University of TCM), Ministry of Education, Fuzhou, China

**Keywords:** Tai Chi, quality of life, body mass index, meta-analysis, type 2 diabetes mellitus

## Abstract

**Background:**

Type 2 diabetes mellitus (T2DM) is a worldwide public health concern with high morbidity and various progressive diabetes complications that result in serious economic expenditure and social burden. This systematic review aims to evaluate the effect of Tai Chi on improving quality of life (QoL), body mass index (BMI) and waist-hip ratio (WHR) in patients with T2DM.

**Method:**

A systematic review and meta-analysis was performed following PRISMA recommendation. Four English databases and three Chinese databases were searched. The PEDro scale was used to assess the methodological quality of including studies. Study inclusion criteria: randomized controlled trials (RCTs) and quasi-experimental studies were included, patients with T2DM that adopted Tai Chi as intervention and QoL, BMI and/or WHR as outcome measurements.

**Results:**

Eighteen trials were included. The aggregated results of seven trials showed that Tai Chi statistically significantly improved QoL measured by the SF-36 on every domains (physical function: MD = 7.73, 95% confidence interval (CI) = 1.76 to 13.71, p = 0.01; role-physical function: MD = 9.76, 95% CI = 6.05 to 13.47, p < 0.001; body pain: MD = 8.49, 95% CI = 1.18 to 15.8, p = 0.02; general health: MD = 9.80, 95% CI = 5.77 to 13.82, p < 0.001; vitality: MD = 6.70, 95% CI = 0.45 to 12.94, p = 0.04; social function: MD = 9.1, 95% CI = 4.75 to 13.45, p < 0.001; role-emotional function: MD = 7.88, 95% CI = 4.03 to 11.72, p < 0.001; mental health: MD = 5.62, 95% CI = 1.57 to 9.67, p = 0.006) and BMI (MD = −1.53, 95% CI = −2.71 to −0.36, p < 0.001) compared with control group (wait list; no intervention; usual care; sham exercise).

**Conclusion:**

Tai Chi could improve QoL and decrease BMI for patients with T2DM, more studies are needed to be conducted in accordance with suggestions mentioned in this review.

## Introduction

Diabetes mellitus (DM) is a chronic endocrine metabolic disorder characterized by hyperglycemia resulting from insulin secretion dysfunction (1). Type 2 DM (T2DM) is the most common form of DM, with over 90% of adults with DM presenting T2DM (2). It is a worldwide public health concern because of its high morbidity and various progressive complications. It was reported that there were approximately 415 million adults with DM, leading to 5.0 million deaths. Moreover, the total global health-related economic expenditure due to DM was about 673 billion US dollars in 2015 (3). The number of adults with DM was predicted to increase to 642 billion by 2040 (3). As a country with the largest population and the largest number of DM patients, the estimated DM prevalence in China was 10.9% (4). The total medical costs due to DM and its complications accounted for over 4% of the total national medical expenses in China (5). T2DM patients are at high risk of experiencing associated cardiovascular diseases, diabetic neuropathy, and kidney diseases (1). Previous studies have emphasized that the quality of life (QoL) in these adults is usually poorer than that of normal controls without DM (6, 7). Both diabetic complications and comorbid conditions could determine the QoL of T2DM patients. Hence, the treatment of DM aims to prevent complications and improve the QoL in these patients.

In the current guidelines, the main strategy to manage T2DM has been intensive glycemic control (8–10). However, a body of evidence has demonstrated that intensive glycemic control increases the risk of severe hypoglycemia, polypharmacy, and side effects (11). Glycemic control should be aligned to each patient’s situations and goals in order to minimize diabetic complications, reduce the economic burden, and improve the QoL. Physical exercise also plays an important protective role in altering the body composition, blood pressure, and glycemic control as a non-pharmaceutical and cost-effective treatment in T2DM patients (12, 13). Observation studies have shown that higher levels of physical exercise could improve the QoL in T2DM patients (14, 15). Furthermore, it has been confirmed that physical exercise reduces morbidity and mortality and increases insulin sensitivity in these patients (12, 13). Tai Chi is a low to moderate intensity, mind-body exercise that originated in China and gained popularity worldwide (16). A national health survey in America reported that Tai Chi was one of the top three most frequently adopted complementary therapies with general effectiveness and no serious adverse events (17). Tai Chi had similar health benefits as that of general exercise in terms of resting energy expenditure, body composition, aerobic fitness, and self-perceived health, but lower energy metabolism levels (18).

QoL reflects an individual’s perception of their physical, psychological, and social status. T2DM has negative effects on both physical and mental states (19). Diabetic peripheral neuropathy can lead to body pain and foot ulcers, even resulting in amputation, which could have a serious negative impact on the QoL (20). A previous systematic review showed that aerobic exercise, resistance exercise, or a combination of both could improve the QoL of patients with T2DM (21). Individuals with T2DM had higher medical expenditures and raising more with increment of body mass index (BMI) than those without T2DM (22). Management of weight-related indicators has been demonstrated to reduce the incidence of T2DM and its complications dramatically (23). Weight management is an important therapeutic strategy in T2DM, and the reduction in BMI has been associated with good metabolic control with appreciable economic benefits (24, 25).

The evidence regarding the effect of exercise on the QoL and weight management in T2DM patients from recent systematic reviews is insufficient (26, 27). Moreover, there were limited studies on the treatment of T2DM with Tai Chi. Only certain reviews and/or meta-analyses have explored the benefits of Tai Chi in T2DM patients (28–31). Although three reviews (28, 29, 31) investigated the outcome of Tai Chi using biomarkers, none of them investigated the outcomes with respect to QoL, BMI, and waist-hip ratio (WHR) together. Zhou et al. (28). investigated its impact on QoL and BMI, while Lee’s review (30) only assessed the impact on QoL. Lee’s review (30) included only three trials of pooled analysis reporting the superior effect of Tai Chi, whereas Zhou’s review (28) reported only three sub-items (physical function, bodily pain, and social function) of the SF-36. Lee’s review was conducted for a relatively long time and did not retrieve Chinese studies due to language or search source limitations (30). Zhou’s review (28) included relatively incomprehensible eligible original studies with several missing studies (32–35). Ongoing research has continuously generated new evidence. This systematic review critically evaluated and synthesized published studies on the effectiveness of Tai Chi in treating T2DM patients by evaluating its impact on QoL, BMI, and WHR when compared with different control groups (e.g., waitlist, no intervention, usual care, and other exercises).

## Materials

### Search Strategy

This systematic review and meta-analysis was performed according to the Preferred Reporting Items for Systematic Review and Meta-analysis. We did not publish a protocol before conducting the systematic review. Ethical approval and patient informed consent were not applicable since all data collection and analysis were based on previously published articles.

Literature searches were performed in seven electronic databases, including PubMed, Web of Science, Embase, Cochrane Library, Chinese National Knowledge Infrastructure (CNKI), Wanfang, and Chinese Science and Technique Journals Database (VIP), from their inception time to February 2020. The language of the searched literature was limited to English and Chinese. The search terms focused on two key terms: “Tai Chi” and “Diabetes Mellitus.” Combinations of Medical Subject Headings and text words using Boolean operators were adopted for search strategy. The reference lists of all relevant studies and reviews were manually searched to identify potentially eligible literature. Gray literatures with full text (e.g., thesis, dissertations) were also included while conference proceedings abstracts were excluded. **Table 1** shows an example of the search strategy used for PubMed.

**Table 1:** Searching strategy in PubMed.

Search	Query
#1	Tai Ji [MeSH Terms] OR Tai-ji [All Fields] OR Tai Chi [All Fields] OR Chi, Tai [All Fields] OR Tai Ji Quan [All Fields] OR Ji Quan, Tai [All Fields] OR Quan, Tai Ji [All Fields] OR Taiji [All Fields] OR Taijiquan [All Fields] OR T’ai Chi [All Fields] OR Tai Chi Chuan [All Fields]
#2	Diabetes Mellitus, Type 2 [MeSH Terms] OR Diabetes Mellitus, Noninsulin-Dependent [All Fields] OR Diabetes Mellitus, Noninsulin Dependent [All Fields] OR Diabetes Mellitus, Ketosis-Resistance [All Fields] OR Diabetes Mellitus, Ketosis Resistance [All Fields] OR Diabetes Mellitus, Non Insulin Dependent [All Fields] OR Diabetes Mellitus, Non-Insulin-Dependent [All Fields] OR Diabetes Mellitus, Slow Onset [All Fields] OR Diabetes Mellitus, Slow-Onset [All Fields] OR Diabetes Mellitus, Maturity-Onset [All Fields] OR Diabetes Mellitus, Maturity Onset [All Fields] OR Diabetes Mellitus, Type II [All Fields] OR Type 2 Diabetes Mellitus [All Fields] OR Type 2 Diabetes [All Fields] OR Diabetes, Type 2 [All Fields] OR NIDDM [All Fields] OR MODY [All Fields]
#3	#1 and #2

### Inclusion and Exclusion Criteria

Both randomized controlled trials (RCTs) and quasi-experimental studies were included to evaluate the effect of Tai Chi in patients with T2DM, regardless of the intervention length. Tai Chi should be performed as a major intervention method. The following experimental comparisons were eligible in our systematic review: Tai Chi vs. control (wait-list, no intervention, usual care, and sham exercise); Tai Chi vs. other exercises (walking, aerobic dancing, aerobic exercise, etc.); and Tai Chi + standard diabetic care vs. standard diabetic care alone. Outcome measurements of the included studies should have covered at least one of the essential assessments of QoL, BMI, or WHR. All instruments measuring QoL were included, such as SF-36 and diabetes-specific quality of life (DSQoL). As important indicators for T2DM patients, BMI and WHR were the most commonly used outcomes to measure body composition (36).

### Data Extraction and Quality Assessment

Two reviewers (SG, YY) independently scanned the titles and abstracts of each identified study in order to exclude irrelevant literature. The full texts were then independently read by the two reviewers to decide whether these studies were consistent with the selection criteria, and detailed data were independently extracted from the selected studies. Standard data-extraction forms adapted from the Cochrane Collaboration model were used to extract information regarding authors, year and language of the studies published, experiment locations, sample size, age, disease duration, characteristics of the intervention group and control group, outcome measures, drop outs, adverse events and main findings. Missing data were requested directly from the original author *via* e-mail if necessary. Discrepancies were discussed to reach a consensus.

Two reviewers (JQ, YC) assessed the selected studies’ methodological quality according to the Physiotherapy Evidence Database (PEDro) scale. The PEDro scale contained the following 11 items: random allocation, concealed allocation, baseline comparability, blinding participants, blinding therapists, blinding assessors, adequate follow-up, intention-to-treat analysis, between-group comparisons, point estimates, and variability. The maximum score on the PEDro scale was 10 points (item 1 was not counted in the total score), wherein a score of 9 to 10 was categorized as excellent quality, 6 to 8 as good quality, 4 to 5 as fair quality, and < 4 as poor quality. Points were only awarded when a criterion was clearly fulfilled according to its instruction (37). The reliability of PEDro scale for assessing the quality of RCTs was “fair” to “good” (37), and it was deemed appropriate for this systematic review. Disagreements were discussed and resolved by a third reviewer (JQ).

### Statistical Analysis

Review Manager 5.3 (RevMan 5.3, The Nordic Cochrane Centre, The Cochrane Collaboration, Copenhagen, Denmark) was used for the pooled analysis. The statistical heterogeneity among the selected studies was examined using a chi-square test and I^2^ value. The heterogeneity was considered low if I^2^ was <50% and high if I^2^ was >50% (38). We conducted data synthesis by using a random effects model, regardless of the level of heterogeneity. The endpoint value was employed for outcome data and expressed as mean difference (MD) or the standard mean difference and the 95% confidence interval (CI) for further data synthesis. For the multiple group studies, the “shared” group was evenly divided into more groups with relatively small sample sizes, and further comparisons were conducted. A p-value <0.05 was considered statistically significant. Subgroup analysis for different intervention comparisons was performed to explore the heterogeneity source. Sensitivity analysis was also conducted to explore the heterogeneity source and check the pooled results’ stability by excluding the selected studies one by one. A funnel plot was used to assess possible publication bias if more than 10 trials were pooled for meta-analysis (39).

## Results

### Literature Search

In total, 718 studies were identified after searching the electronic databases, and 480 studies remained after excluding duplicates. Among these studies, 418 records were removed due to the bias of titles and abstracts, and 44 records were excluded after screening the full texts.

**Figure 1:**
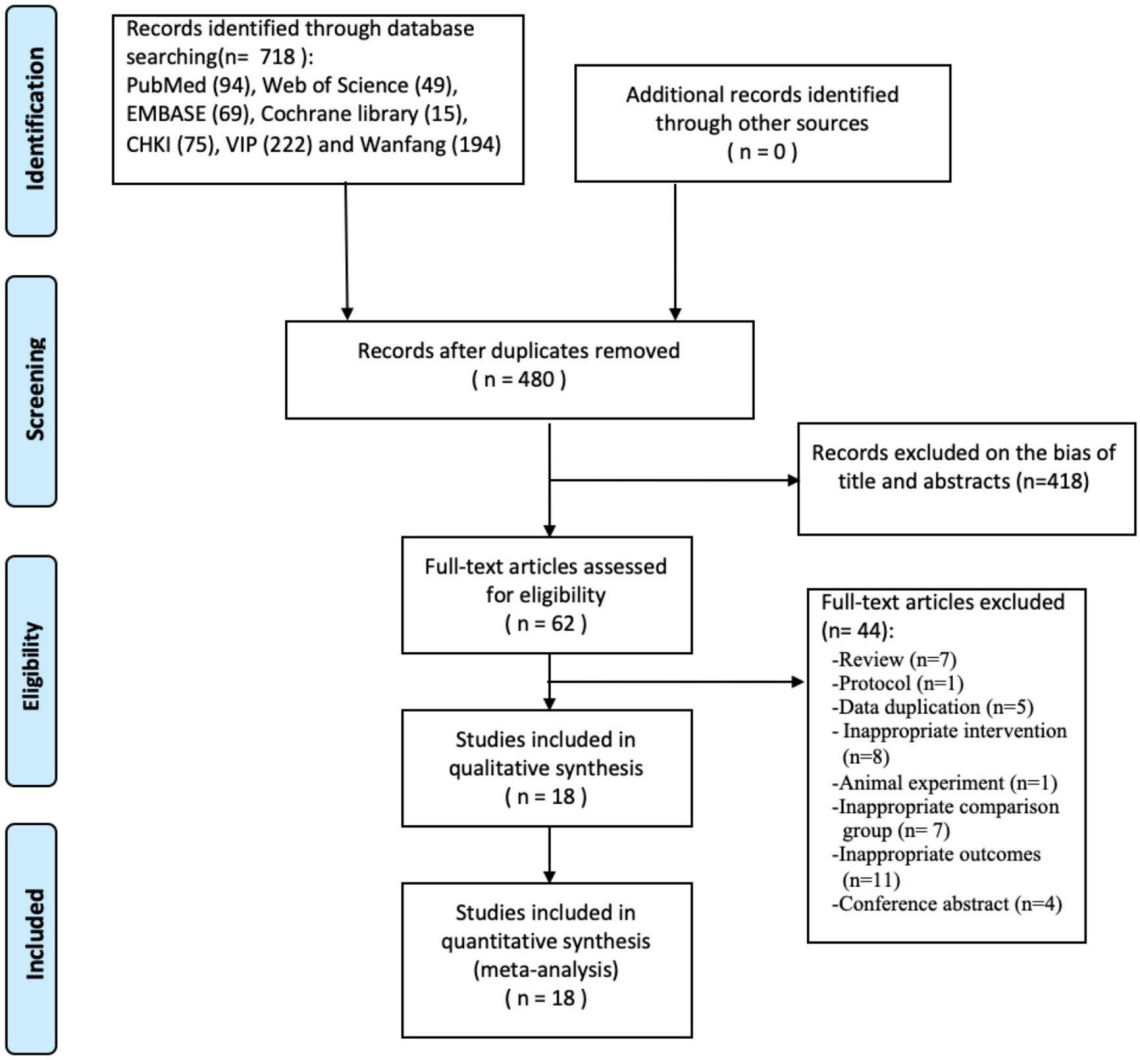
Flowchart of the study selection.

Finally, 18 clinical trials, including three quasi-experimental studies (40–42) and 15 RCTs, were retrieved for systematic review and meta-analysis (**Figure 1**). Of the 18 articles, five (32, 35, 40, 43, 44) were published in English, and 13 (33, 34, 41, 42, 45–53) were in Chinese.

### Characteristic of the Included Studies

In this review, 18 studies involving 1418 participants (94 attritions) were included, of which two trials (35, 44) were conducted in Australia, and one trial each in South Korea (40) and Thailand (43), and the remaining 14 were in China. The sample size of these studies ranged from 16 to 216, and the mean age ranged from 47 to 70 years. The average disease duration ranged from 0.5 to 18 years. Among the included trials, 11 studies (33–35, 40, 42, 44–47, 49, 52) compared the experimental intervention between Tai Chi and control (waitlist, no intervention, usual care, and sham exercise), six (32, 34, 41, 48, 50, 51) compared Tai Chi with other exercises (walking, dancing, aerobic exercise, etc.), and two (43, 53) compared Tai Chi + standard diabetic care with standard diabetic care alone. Fifteen articles (32–35, 41, 43–48, 50–53) adopted Tai Chi Quan with various styles, including 24 forms, Chen style, Yang and Sun style, and Lin style, whereas one article adopted Tai Chi sticks (52) and one adopted Tai Chi balls (33). For Tai Chi intervention, the training time ranged from 30-120 min per day, frequencies ranged from one to seven times per week, and treatment duration ranged from 12 to 24 weeks. For outcome measurements, eight studies reported outcomes with respect to QoL, 11 studies reported for BMI, and four studies reported for WHR. The main characteristics of all included trials are shown in **Table 2**.

**Table 2:** The characteristics of all including trials.

Reference	Location(Language)	Sample size	Mean age or age range	Disease duration	Intervention Group time/per week/duration	Control group	Outcome measure	Attrition	AE	Main findings (p value of intergroup/intragroup difference)
Shen XY et al. (48)	China (Chinese)	TG: 54 CG: 54	TG: 67.8 ± 5.1 CG: 66.2 ± 4.6	TG: 9.6 ± 6.8 y CG: 7.6 ± 5.9 y	24 form Tai Chi 60 min/3/12 weeks	Walking 60 min/3/12 weeks	DSQOL	TG: 2 CG: 5	NR	DAQOL: TG showed significant improvement in DAQOL compared to CG(p<0.05).
Wu F et al. (46)	China (Chinese)	TG: 20 CG: 20	TG: 51.3 ± 7.9 CG: 52.4 ± 5.5	TG: 1.35 ± 0.62 y CG: 1.36 ± 0.71 y	24 form Tai Chi 60 min/3/24 weeks	No intervention	SF-36	NR	NR	SF-36: TG showed significant improvements in PF/RP/GH/RE/SF/MH compared to CG (p<0.05).
Lam P et al. (44)	Australia (English)	TG: 28 CG: 25	TG: 63.2 ± 8.6 CG: 60.7 ± 12.2	> 6 m	Yang and Sun style 20 form Tai Chi 60 min/3/12 weeks; 60 min/1/further 12 weeks	Wait list	SF-36	TG: 7 CG: 3	NR	SF-36: TG showed significant improvements in PF/SF/GH from baseline to post-treatment (all p<0.05). No significant difference in all SF-36 domains between two groups (p>0.05).
Li ZB et al. (34)	China (Chinese)	TG: 54 CG(a): 54 CG(b): 54 CG(c): 54	TG: 54.21 ± 9.47 CG(a): 50.42 ± 9.68 CG(b): 51.62 ± 7.83 CG(c): 52.69 ± 8.37	TG: 83.42 ± 43.52 m CG(a): 72.18 ± 39.57 m CG(b): 75.74 ± 42.39 m CG(c): 86.65 ± 49.72 m	24 form Tai Chi 30 min/7/12 weeks	CG(a): Baduanjin CG(b): Aerobic Exercise (brisk walking. etc) CG(c): No intervention	BMI	TG: 11 CG(a): 4 CG(b): 6 CG(c): 10	NR	BMI: TG showed significant improvement in BMI from baseline to post-treatment (p<0.05). Intergroup comparison was NR.
Meng E (49)	China (Chinese)	TG: 100 CG: 100	68. ± 3.2	2-23 y	Tai Chi 3 months	Wait list	SF-36	NR	NR	SF-36: TG showed significant improvement in GH and SF-36 total score compared to CG (p<0.05).
Wang P et al. (47)	China (Chinese)	TG: 34 CG: 30	TG: 48.24 ± 10.06 CG: 47.86 ± 11.12	TG: 1-18 y CG: 1-17 y	24 form Tai Chi 45–60 min/5-7/24 weeks	No intervention	SF-36	NR	NR	SF-36: TG showed significant improvement in RE (P<0.01), PF/RP/BP/GH/VT/SF (p<0.05), and SF-36 total score/PCS/MCS (p<0.01) compared to CG.
Wei DL et al. (33)	China (Chinese)	TG: 26 CG: 26	56.0 ± 7.2	0.5-3 y	Tai Chi ball 36 form 60 min/6/12 weeks	Usual care	BMI; WHR	NR	NR	BMI/WHR: TG showed significant improvement in BMI/WHR from baseline to post-treatment(p<0.05). Intergroup comparison was NR.
Ahn S (40)	South Korea (English)	TG: 30 CG: 29	TG: 66.05 ± 6.42 CG: 62.73 ± 7.53	TG: 12.30 ± 8.81 y CG: 13.00 ± 10.03 y	Tai Chi 60 min/2/12 weeks	Usual care	SF-36	TG: 10 CG: 10	NR	SF-36: TG showed significant improvement in PF/BP/RP/RE/SF compared to CG (p<0.05). No significant difference in SF-36 subcomponents (PCS/MCS) between two groups (p>0.05).
Chen SC et al. (32)	China (English)	TG: 62 CG: 55	TG: 59.1 ± 6.2 CG: 57.4 ± 5.8	TG: 8.5 ± 3.5 y CG: 7.8 ± 3.1 y	Chen style 99 form Tai Chi 60 min/3/12 weeks	Aerobic dancing 60 min/3/12 weeks	BMI	TG: 6 CG: 7	NR	BMI: TG showed significant improvement in BMI compared to CG (p=0.017).
Trang T et al. (35)	Australia (English)	TG: 18 CG: 20	TG: 60 ± 8 CG: 65 ± 8	TG: 8.5 y CG: 9.0 y	Yang and Sun style Tai Chi, 60 min/2/12 weeks	Sham exercise	SF-36	TG: 1	Y	SF-36: TG showed significant improvement in SF compared to CG (p=0.04).
Cai H (42)	China (Chinese)	TG: 27 CG: 28	TG: 65.54 CG: 64.51	TG: 4.93 y CG: 5.44 y	Tai Chi 30 min/3/12 weeks	Wait list	BMI	CG:5	NR	BMI: TG showed significant improvement in BMI compared to CG (p=0.004).
Chen ZC (52)	China (Chinese)	TG: 30 CG: 30	TG: 60.71 ± 7.06 CG: 61.14 ± 5.27	TG: 5.58 ± 2.77 y CG: 6.00 ± 2.58 y	Tai Chi stick 60 min/4/12 weeks	No intervention	BMI; WHR; SF-36	TG: 2	NR	BMI/WHR: TG showed significant improvement in BMI/WHR from baseline to post-treatment(p<0.05). SF-36: TG showed significant improvement in all SF-36 domains compared to CG (all p<0.05).
Li HC et al. (50)	China (Chinese)	TG: 50 CG: 50	TG: 62.91 ± 2.48 CG: 63.27 ± 2.86	TG: 7.83 ± 2.16 y CG: 8.14 ± 3.19 y	Chen style Tai Chi 40–50 min/7/24 weeks	Aerobic Exercise	BMI; WHR	NR	NR	BMI/WHR: No significant difference in BMI and WHR between two groups (p>0.05).
Bao QW et al. (53)	China (Chinese)	TG: 58 CG: 49	TG: 60.4 ± 6.9 CG: 68.4 ± 7.1	NR	Tai Chi + standard diabetic care 120 min/14/24 weeks	Standard diabetic care	BMI; WHR	NR	NR	BMI/WHR: TG showed significant improvement in BMI and WHR compared to CG (p<0.05).
Zhao G et al. (45)	China (Chinese)	TG: 8 CG: 8	TG: 54.75 ± 6.09 CG: 52.38 ± 7.65	NR	Chen style 60 min/7/16 weeks	No intervention	BMI	NR	NR	BMI: No significant difference in BMI between two groups (p>0.05).
Youngwan-chsetha S et al. (43)	Thailand (English)	TG: 34 CG: 35	TG: 60.71 ± 7.06 CG: 61.14 ± 5.27	TG: 2.47 ± 1.24 y CG: 2.78 ± 1.18 y	Lin Housheng style Tai Chi + standard diabetic care 50 min/5/12 weeks	Standard diabetic care	BMI	TG: 2 CG: 3	NR	BMI: No significant difference in BMI between two groups (p=0.10).
Kan Y et al. (51)	China (Chinese)	TG: 26 CG: 22	52 ± 6.7	>3 y	24 form Tai Chi 60 min/7/12 weeks	Aerobic Exercise	BMI	NR	NR	BMI: TG showed significant improvement in BMI compared to CG (p<0.05).
Wang JH et al. (41)	China (Chinese)	TG: 10 CG: 6	60–70	>2 y	24 form Tai Chi 60 min/7/12 weeks	Aerobic Exercise	BMI	NR	NR	BMI: TG showed significant improvement in BMI compared to CG (p<0.05).

TG, Tai Chi Group; CG, control group; AE, adverse events; y, year; m, month; DSQOL, diabetes-specific quality of life; SF-36, Medical Outcomes Study Short Form-36; BMI, body mass index; WHR, waist-hip ratio; NR, not reported; SF-36 domains (PF, physical functioning; RP, role-physical; BP, bodily pain; GH, general health; VT, vitality; SF, social functioning; RE, role-emotional; MH, mental health; PCS, physical component summary, MCS=mental component summary).

### Methodological Quality

The methodological quality of the included trials ranged from 3 to 7 points, and the mean PEDro scale score was 5.3. Eight studies (33, 35, 43, 45–47, 49, 53) were of “good” quality, nine studies (32, 34, 41, 42, 44, 48, 50–52) were of “fair” quality, and one study (40) was of “poor” quality (**Table 3**). Blinding of participants, therapists, and evaluators was not conducted in most trials. Three trials (41, 50, 51) did not report whether the baseline information was comparable. Only one trial (43) reported concealed allocation, and one trial (40) showed >15% attrition. Eight trials (32, 34, 40, 42–44, 48, 52) did not use intention-to-treat analysis. The remaining items were positive in all trials.

**Table 3:** PEDro score for methodological quality assessment of including studies.

Reference	Item 1	Item 2	Item 3	Item 4	Item 5	Item 6	Item 7	Item 8	Item 9	Item 10	Item 11	score
Shen XY et al. (48)	1	1	0	1	0	0	0	1	0	1	1	5/10
Wu F et al. (46)	1	1	0	1	0	0	0	1	1	1	1	6/10
Lam P et al. (44)	1	1	0	1	0	0	0	1	0	1	1	5/10
Li ZB et al. (34)	1	1	0	1	0	0	0	1	0	1	1	5/10
Meng E (49)	1	1	0	1	0	0	0	1	1	1	1	6/10
Wang P et al. (47)	1	1	0	1	0	0	0	1	1	1	1	6/10
Wei DL (33)	1	1	0	1	0	0	0	1	1	1	1	6/10
Ahn S (40)	1	0	0	1	0	0	0	0	0	1	1	3/10
Chen SC et al. (32)	1	1	0	1	0	0	0	1	0	1	1	5/10
Trang T et al. (35)	1	1	0	1	0	0	0	1	1	1	1	6/10
Cai H (42)	1	0	0	1	0	0	0	1	0	1	1	4/10
Chen ZC (52)	1	1	0	1	0	0	0	1	0	1	1	5/10
Li HC et al. (50)	1	1	0	0	0	0	0	1	1	1	1	5/10
Bao QW et al. (53)	1	1	0	1	0	0	0	1	1	1	1	6/10
Zhao G et al. (45)	1	1	0	1	0	0	0	1	1	1	1	6/10
Youngwanichsetha S et al. (43)	1	1	1	1	0	0	1	1	0	1	1	7/10
Kan Y et al. (51)	1	1	0	0	0	0	0	1	1	1	1	5/10
Wang JH et al. (41)	1	0	0	0	0	0	0	1	1	1	1	4/10

Item 1, eligibility criteria; item 2, random allocation; item 3, concealed allocation; item 4, similar baseline; item 5, subjected blinded; item 6, therapists blinded; item 7, assessors blinded; item 8, < 15% dropouts; item 9, intention-to-treat analysis; item 10, between-group comparison; item 11, point measures and variability data; 1, described explicitly and in details; 0, unclear, inadequately described.

### Effect of Tai Chi on Body Mass Index

**Figure 2:**
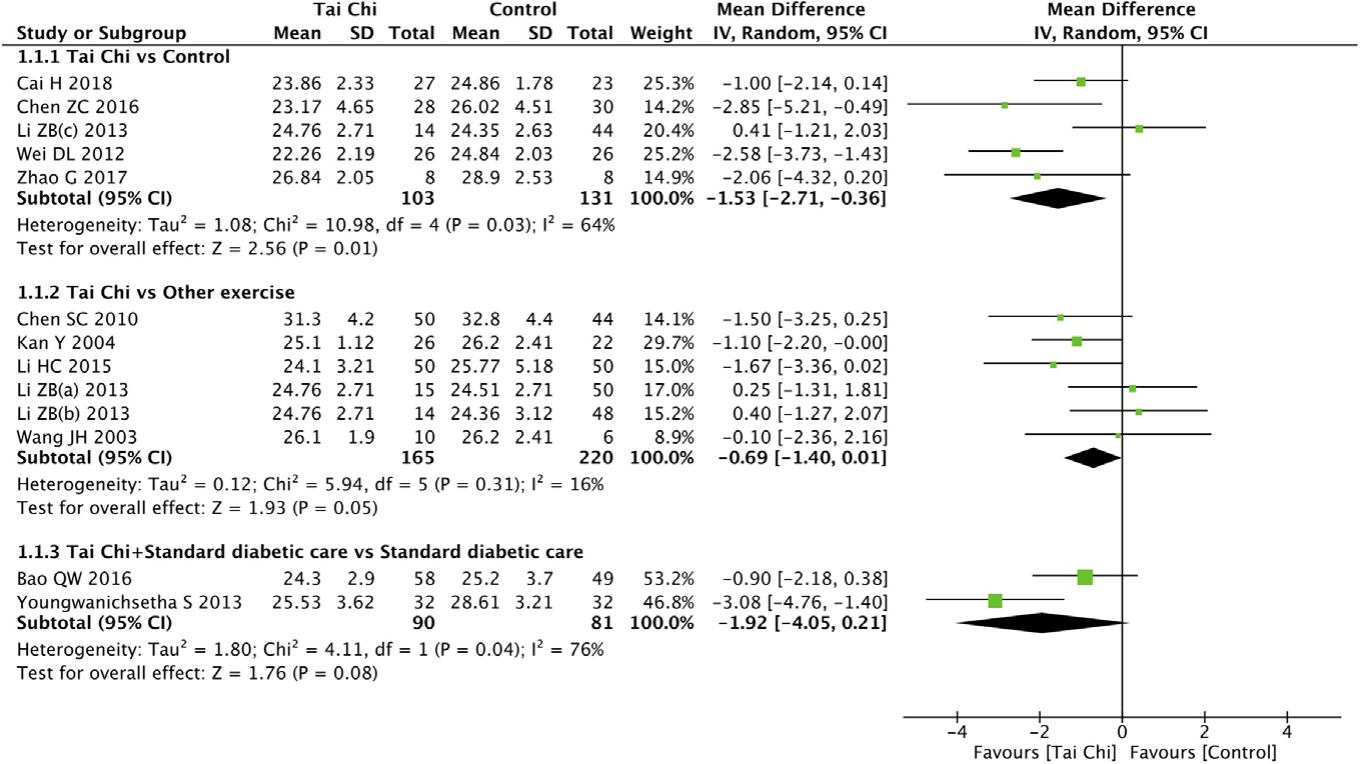
Forest plot of effect for Tai Chi in BMI.

Eleven trials (32–34, 41–43, 45, 50–53) including 13 comparisons explored the effect of Tai Chi on BMI. When compared to the controls (wait list, no intervention, usual care, and sham exercise), the Tai Chi group showed significant improvements in BMI (n = 234; MD = −1.53; 95% CI, −2.71 to −0.36; p = 0.01; heterogeneity, I^2^ = 64%, p = 0.03; **Figure 2**). However, when compared to the other exercises (walking, dancing, and aerobic exercise), the Tai Chi group did not show significant improvements in BMI (n = 385; MD = −0.69; 95% CI, −1.40 to 0.01; p = 0.05; heterogeneity, I^2^ = 16%, p = 0.31; **Figure 2**). Similarly, in the analysis of Tai Chi + standard diabetic care vs. standard diabetic care alone, the former showed no significant improvements in BMI (n = 171; MD = −1.92; 95% CI, −4.05 to 0.21; p = 0.08; heterogeneity, I^2^ = 76%, p = 0.04; **Figure 2**).

### Effect of Tai Chi on Waist-Hip Ratio

**Figure 3:**
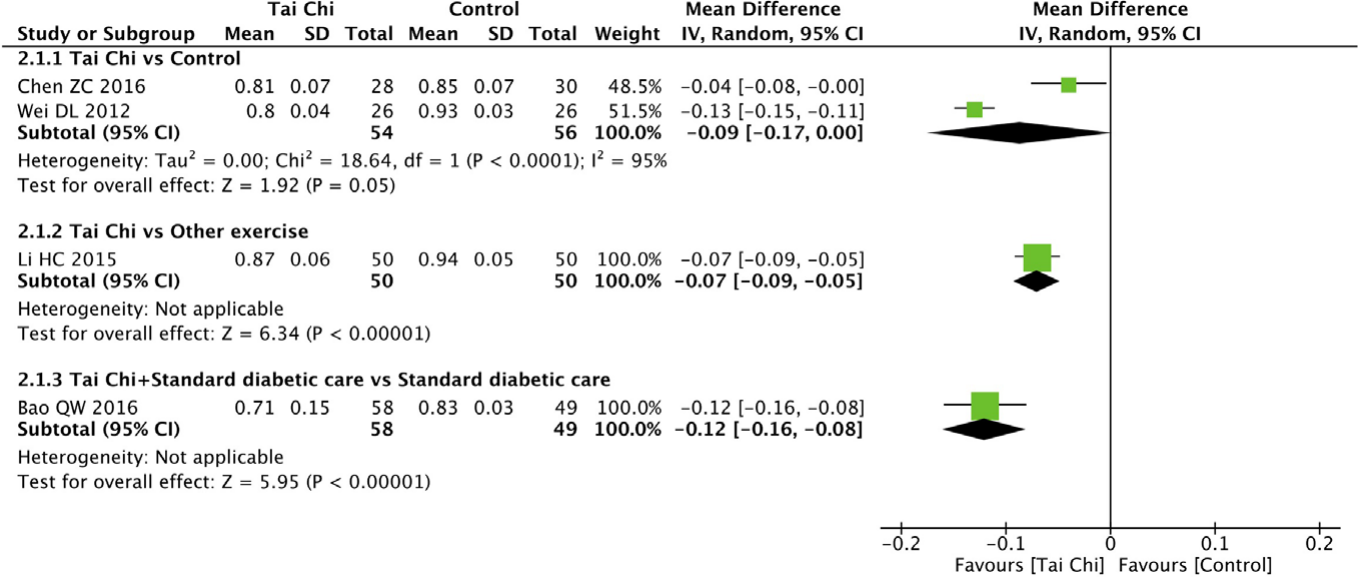
Forest plot of effect for Tai Chi in WHR.

Four trials (33, 50, 52, 53) explored the effect of Tai Chi on WHR. In the analysis of Tai Chi vs. control (wait list, no intervention, usual care, and sham exercise), the former did not show significant improvements in WHR (n = 110; MD = −0.09; 95% CI, −0.17 to 0.00; p = 0.05; heterogeneity, I^2^ = 95%, p < 0.001; **Figure 3**). In the analysis of Tai Chi vs. other exercises and Tai Chi + standard diabetic care vs. standard diabetic care alone, one trial showed that Tai Chi significantly improved WHR in both comparisons (n = 100; MD = −0.07; 95% CI, −0.09 to −0.05; p < 0.001; **Figure 3**; and n = 107; MD = −0.12; 95% CI, −0.16 to −0.08; p < 0.001; **Figure 3**, respectively).

### Effect of Tai Chi on the Quality of Life

**Figure 4:**
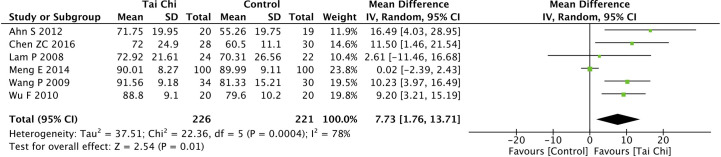
Forest plot of effect for Tai Chi in SF-36 (PF).

**Figure 5:**
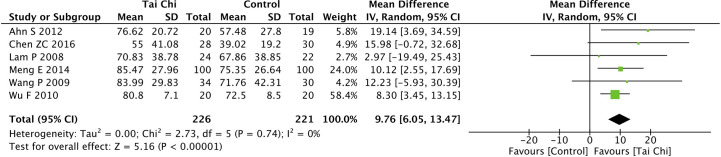
Forest plot of effect for Tai Chi in SF-36 (RP).

**Figure 6:**
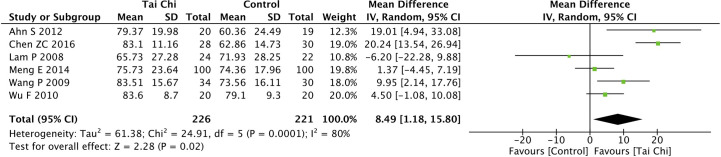
Forest plot of effect for Tai Chi in SF-36 (BP).

**Figure 7:**
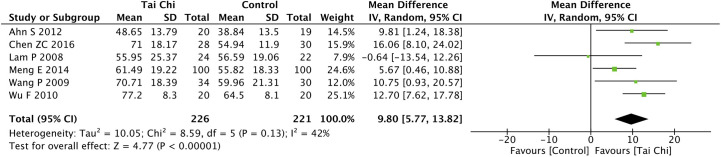
Forest plot of effect for Tai Chi in SF-36 (GH).

**Figure 8:**
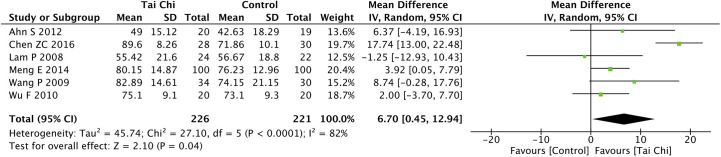
Forest plot of effect for Tai Chi in SF-36 (VT).

**Figure 9:**
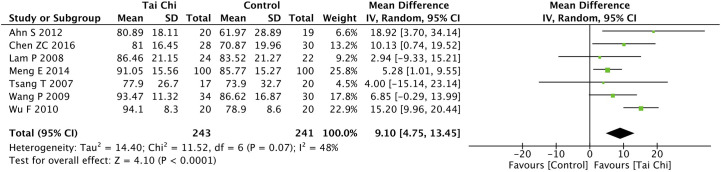
Forest plot of effect for Tai Chi in SF-36 (SF).

**Figure 10:**
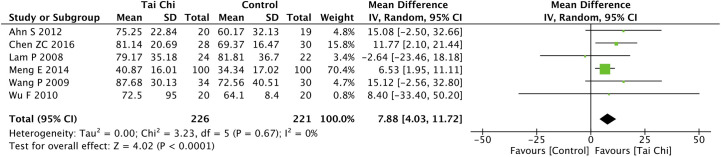
Forest plot of effect for Tai Chi in SF-36 (RE).

**Figure 11:**
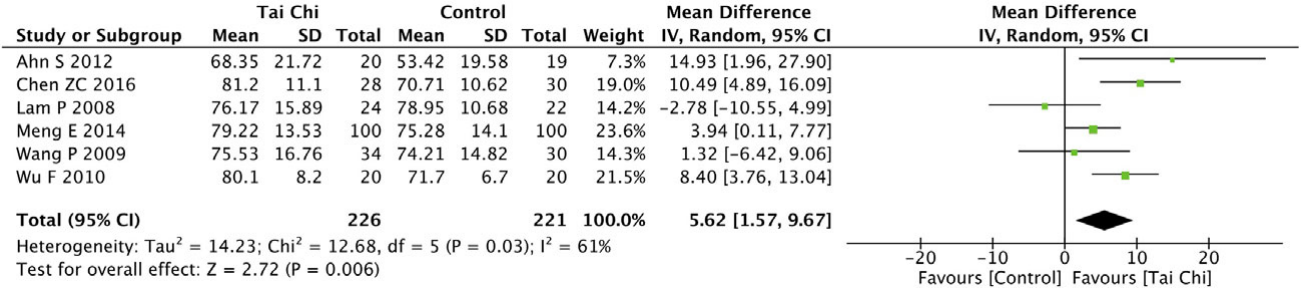
Forest plot of effect for Tai Chi in SF-36 (MH).

Eight trials (35, 40, 44, 46–49, 52) assessed the effect of Tai Chi on QoL. One trial (48) that compared Tai Chi with walking used DSQoL as an assessment tool, whereas the others used SF-36 to compare the effect of Tai Chi with that of the control group (wait list, no intervention, usual care, and sham exercise). For the DSQoL, the trial demonstrated favorable effects of Tai Chi when compared with walking. The SF-36 contains eight domains: physical functioning (PF); role-physical function (RP); body pain (BP); general health (GH); vitality (VT); social functioning (SF); role-emotional function (RE); and mental health (MH). The meta-analysis of seven trials (35, 40, 44, 46, 47, 49, 52) showed that Tai Chi significantly improved the scores of all SF-36 sub-items: PF (n = 447; MD = 7.73; 95% CI, 1.76 to 13.71; p = 0.01; heterogeneity, I^2^ = 78%, p < 0.001; **Figure 4**); RP (n = 447; MD = 9.76; 95% CI, 6.05 to 13.47; p < 0.001; heterogeneity, I^2^ = 0%, p = 0.74; **Figure 5**); BP (n = 447; MD = 8.49; 95% CI, 1.18 to 15.8; p = 0.02; heterogeneity, I^2^ = 80%, p < 0.001; **Figure 6**); GH (n = 447; MD = 9.80; 95% CI, 5.77 to 13.82; p < 0.001; heterogeneity, I^2^ = 42%, p = 0.13; **Figure 7**); VT (n = 447; MD = 6.70; 95% CI, 0.45 to 12.94; p = 0.04; heterogeneity, I^2^ = 82%, p < 0.001; **Figure 8**); SF (n = 484; MD = 9.1; 95% CI, 4.75 to 13.45; p < 0.001; heterogeneity, I^2^ = 48%, p = 0.07; **Figure 9**); RE (n = 447; MD = 7.88; 95% CI, 4.03 to 11.72; p < 0.001; heterogeneity, I^2^ = 0%, p = 0.67; **Figure 10**); and MH (n = 447; MD = 5.62; 95% CI, 1.57 to 9.67; p = 0.006; heterogeneity, I^2^ = 61%, p = 0.03; **Figure 11**).

Sensitivity analysis was conducted to explore the possible sources of heterogeneity and evaluate the stability of the pooled results by removing these studies one by one. Heterogeneity was reduced to 0% by removing Meng’s trial (49), Chen’s trial (52), and Wu’s trial (46) with respect to PF, VT, and SF, respectively. Nonetheless, the pooled results of all SF-36 sub-items appeared relatively consistent.

### Adverse Events

Only Tsang’s trial (35) reported adverse events in which one participant in the Tai Chi group presented exercise intolerance due to pain and fatigue. This participant had pre-existing spinal stenosis but was not symptomatic during the process of screening and baseline assessment, and quit the project after attending one Tai Chi exercise session in Tsang’s trial (35).

## Discussion

The objective of this systematic review and meta-analysis was to evaluate the effect of Tai Chi in T2DM patients on their QoL, BMI, and WHR. The pooled results showed that Tai Chi could significantly improve the QoL of T2DM patients with respect to all SF-36 sub-items (PF, RP, BP, GH, VT, SF, RE, and MH) when compared to the control group (waitlist, no intervention, usual care, and sham exercise). The Tai Chi group showed a significant decrease in the T2DM severity of patients in terms of BMI when compared to the control group. Limited studies have shown that Tai Chi could reduce the WHR in T2DM patients when compared with other exercises or as an adjunctive treatment.

This systematic review and meta-analysis aimed to update the evidence by including recent clinical trials of Tai Chi in patients with T2DM. Compared to two previous related reviews (28, 30), we identified three and five new trials that examined the effect of Tai Chi on the QoL and BMI of T2DM patients, respectively. The results of our review were consistent with those of previous reviews (28, 30). Lee’s review narratively reported the superior effects of Tai Chi on QoL when compared with the control group (no treatment or waitlist) (30). Zhou’s review suggested that Tai Chi could improve QoL in the domains of PF, BP, and SF by synthesizing the results of five trials (28). Our review showed that Tai Chi significantly improved the scores of all SF-36 sub-items in T2DM patients when compared with the control group (wait list, no intervention, usual care, and sham exercise) by synthesizing the results of seven trials.

Tai Chi has become popular worldwide as an important branch of traditional Chinese mind-body exercise. The major characteristics of Tai Chi include mind concentration with breath control, full-body exercise in a semi-squat position, and continuous spiral body movements (16). During the process of Tai Chi training, body movements and deep diaphragmatic breathing are integrated to achieve a harmonious balance between the mind and body to facilitate internal energy flow (16). Previous research has confirmed the significant benefits of various Tai Chi styles in health promotion. Moreover, Tai Chi practice could improve the muscular strength, aerobic capacity, balance, QoL, and psychological well-being (16, 54–57). It has significant benefits on physical, psychological, and social functions.

Previous studies have concluded that exercise is beneficial for DM patients (58–60). Furthermore, related reviews (30, 31) have shown that Tai Chi training modulates the blood pressure, triglyceride, high-density lipoprotein cholesterol, serum malondialdehyde, and C-reactive protein in DM patients. Several studies (32, 40, 44) reported that Tai Chi training improved balance, neuropathic symptoms, and some dimensions of QoL. In total, eight trials were selected to assess the effect of Tai Chi on the QoL of T2DM patients in this review. Seven of them used SF-36 as the assessment tool, and the comparisons were made with the control group (waitlist, no intervention, usual care, and sham exercise). The pooled results revealed that T2DM patients presented statistically significant differences in all dimensions of SF-36 in the Tai Chi as compared to the control group. Minimal clinically important difference was considered as the smallest improvement of symptoms in a score of assessment tools that patients perceived as beneficial.

In a previous study (61, 62), the minimal clinically important differences for eight SF-36 domains were defined as ≥5 points. The pooled results of our review demonstrated that the two SF-36 domains of GH and RP were both statistically and clinically significant. The other six SF-36 domains (PF, BP, VT, SF, RE, and MH) were statistically significant and possibly clinically significant. The lower limits of 95% CI in the two SF-36 domains of GH and RP were greater than the minimal clinically important difference, but not in the other six SF-36 domains of PF, BP, VT, SF, RE, and MH. Heterogeneity was reduced to 0% by removing Meng’s trial (49), Chen’s trial (52), Wu’s trial (46) for PF, VT, and SF, respectively. The sources of heterogeneity in the aspects of PF, VT, and SF could be the larger sample size (n = 200) in Meng’s trial (49), different Tai Chi style training (Tai Chi stick) in Chen’s trial (52) and longer treatment duration (24 weeks) in Wu’s trial (46) when compared with the other trials. Nonetheless, the pooled results of all SF-36 sub-items appeared relatively consistent.

Tai Chi also showed significant benefits in BMI when compared to the control group, which was consistent with previous literature (28). In addition, the pooled result of this review showed that the Tai Chi group had a BMI reduction of 1.53 points as compared to the control group. The result of BMI reduction in this review could achieve clinical significance, indicating the beneficial effect of Tai Chi in patients with T2DM (63). On the other hand, no significant improvements were noted in the Tai Chi group when compared with other exercises and in the Tai Chi + standard diabetic care group when compared with standard diabetic care alone. Tai Chi and exercise may have similar mechanisms for managing weight in T2DM patients. It is widely accepted that exercise could modulate insulin-dependent and insulin-independent mechanisms, and regular long-term exercises would involve “over crosstalk” that could mediate the related systemic effects on glycated hemoglobin, blood glucose levels, blood pressure, and serum lipid profiles (64). Only one trial showed that the WHR improved significantly with Tai Chi when compared with other exercises and with Tai Chi + standard diabetic care when compared with standard diabetic care alone. Interestingly, the pooled results of two trials showed no significant improvements in WHR in the comparison between the Tai Chi and control groups. The aggravated results had low reliability, possibly due to limited studies and a small sample size. Larger samples and high-quality studies are needed to identify the effect of Tai Chi on weight management in T2DM patients. Most trials did not systematically assess intervention safety. Furthermore, they were poorly described, and only one study reported adverse events. Tai Chi seems to be a safe alternative therapy for T2DM patients.

This systematic review and meta-analysis had some limitations. First, 14 of 18 included studies were conducted in China, and the other four trials were conducted in Australia, Korea, and Thailand. Thirteen studies were published in Chinese and five in English. Second, only one trial (43) reported conceal allocation and assessor blinding. It was impractical to blind the participants and Tai Chi supervisors in all trials. Third, the methodological quality was rated as low (3/10) in one trial (40). There were three quasi-experimental trials and 15 RCTs in our review. The quasi-experimental trials may have had confounding biases due to poor methodological quality. Fourth, many studies had a small sample size. Half of the included studies reported dropout conditions, and one trial (40) showed a high dropout rate of 33.9%. It seems suspicious that nine trials conducted in China and published in Chinese had zero dropouts. Fifth, the style, time, frequency, and duration of treatment of Tai Chi were variable.

For future research, a larger sample size and good methodological quality studies are needed to explore the effect of Tai Chi in T2DM patients. The intervention intensity should be quantitatively measured. The exercise protocols of both the experimental group and control group should be described in detail so that other researchers can reproduce the intervention protocols. Tai Chi participants should be taught to familiarize themselves with Tai Chi movements in several training sessions.

The findings of this systematic review and meta-analysis revealed positive evidence regarding the effectiveness of Tai Chi in improving the QoL, BMI, and WHR of T2DM patients. Various types of Tai Chi can be applied to T2DM patients aged between 50 and 70 years. The minimum valid training duration of Tai Chi for T2DM patients is about 12 weeks. The recommended Tai Chi training frequency is at least thrice a week and 30 to 60 min per training session. Tai Chi could be an alternative for physical activity in T2DM patients to improve the QoL and for weight management.

## Conclusion

Tai Chi showed benefits in T2DM patients by improving the QoL and BMI when compared with controls. As a safe, cost-effective, and convenient mind-body exercise, Tai Chi might be recommended for T2DM patients as an alternative for physical activity. Future research should be conducted with reference to the aforementioned suggestions.

## Data Availability Statement

All datasets generated for this study are included in the article/supplementary material.

## Author Contributions

Conceptualization: JQ, LC, and JT. Methodology: JQ, ZL. Formal analysis: JQ and YC. Data curation: SG and YY. Writing—original draft preparation: JQ. Writing—review and editing: YX, JW, JH. Supervision: LC and JT. All authors contributed to the article and approved the submitted version.

## Funding

This study was supported by a grant from the Key Research and Development project funded by the Ministry of Science and Technology of the People’s Republic of China (grant no. 2019YFC1710301) and the Science and technology platform construction project of Fujian science and Technology Department (grant no. 2018Y2002).

## Conflict of Interest

The authors declare that the research was conducted in the absence of any commercial or financial relationships that could be construed as a potential conflict of interest.
